# Comprehensive Metabolite Identification of Genipin in Rats Using Ultra-High-Performance Liquid Chromatography Coupled with High Resolution Mass Spectrometry

**DOI:** 10.3390/molecules28176307

**Published:** 2023-08-29

**Authors:** Zhifeng Cui, Zhe Li, Weichao Dong, Lili Qiu, Jiayu Zhang, Shaoping Wang

**Affiliations:** 1 School of Pharmacy, Binzhou Medical University, Yantai 264003, China; 2 Binzhou Hospital of Traditional Chinese Medicine, Binzhou 256600, China; 3 College of Pharmacy, Shandong University of Traditional Chinese Medicine, Jinan 250300, China; 4 School of Medical Technology, Binzhou Vocational College, Binzhou 256600, China

**Keywords:** genipin, UHPLC-HRMS, metabolites, data-mining method, fecal fermentation

## Abstract

Genipin, an aglycone of geniposide, is a rich iridoid component in the fruit of *Gardenia jasminoides* Ellis and has numerous biological activities. However, its metabolic profiles in vivo and vitro remain unclear. In this study, an effective analytical strategy based on ultra-high-performance liquid chromatography-high resolution mass spectrometry (UHPLC-HRMS) in positive and negative ion modes was developed to analyze and identify genipin metabolites in rat urine, blood, feces, and fecal fermentation in combination with many methods including post-collection data mining methods, high-resolution extracted ion chromatography (HREIC), and multiple mass defect filtering (MMDF). Simultaneously, the metabolites of genipin in vivo were verified by fecal fermentation of SD rats at different times. Finally, based on information such as reference substances, chromatographic retention behavior, and accurate mass determination, a total of 50 metabolites (including prototypes) were identified in vivo. Among them, 7, 31 and 28 metabolites in vivo were identified in blood, urine, and feces, respectively. Our results showed that genipin could generate different metabolites that underwent multiple metabolic reactions in vivo including methylation, hydroxylation, dehydroxylation, hydrogenation, sulfonation, glucuronidation, demethylation, and their superimposed reactions. Forty-six metabolites were verified in vitro. Meanwhile, 2 and 19 metabolites identified in blood and urine were also verified in fecal fermentation at different times. These results demonstrated that metabolites were produced in feces and reabsorbed into the body. In conclusion, the newly discovered metabolites of genipin can provide a new perspective for understanding its pharmacological effects and build the foundation for thee toxicity and safety evaluations of genipin.

## 1. Introduction

Genipin, an aglycone of geniposide, is widely present in the fruits of *Gardenia jasminoides* Ellis [1]. It has been reported to have numerous biological activities including anti-inflammatory [2], anti-tumor [3], anti-coagulant [4], anti-hypertensive [5], and hypoglycemic activities [6]. In addition, many substances that can exert pharmacological activities in vivo are often not the prototype drug itself but its metabolites. However, metabolites in vivo are often difficult to detect and are easily hidden by the background noise generated by diverse endogenous metabolites. In the past few decades, with the development of analytical instruments, ultra-high-performance liquid chromatography-high-resolution mass spectrometry (UHPLC-HRMS), developed based on high-performance liquid chromatography-mass spectrometry (HPLC-MS), has exhibited excellent sensitivity in the identification of drug metabolites. Simultaneously, a comprehensive and effective analysis method is an important prerequisite for the identification of drug metabolites. Thus, a series of post-acquisition data-mining methods have emerged to obtain information from high-resolution mass spectrometry and multistage mass spectrum datasets. Among them, high-resolution extracted ion chromatography (HREIC) [7], multiple mass defect filtering (MMDF), neutral loss filtering (NLF), and diagnostic product ions (DPIs) could be applied to the detection and identification of metabolites [8]. In our previous study, the metabolites and metabolic pathways in vivo of geniposide, which is the glucoside of genipin, was clearly revealed [9]. However, the metabolic behavior of genipin in vivo has not been fully elucidated. Studies have shown that intestinal flora is an important factor causing changes in the human body environment, and intestinal microecological disorders may cause a series of diseases [10,11,12,13]. Fresh feces is rich in a large number of intestinal microbial species, which can simulate the intestinal environment in vivo, corresponding to biological transformation to a certain extent. Therefore, genipin in vitro fecal fermentation can well simulate the intestinal environment to verify the metabolites in vivo.

Although the previous study already reported 10 metabolites of genipin in Sprague-Dawley (SD) rats and revealed the metabolism of genipin in vivo, the metabolic pathways in vivo were unclearly explained due to the lack of a reasonable and scientific analytical strategy [14]. In this study, the UHPLC-HRMS-based strategy, which is the full scan-dynamic exclusion data-acquisition method coupled with multiple data-mining techniques including MMDF, HREIC and DPIs, was applied to improve the accuracy of metabolite identification, and was established to identify the genipin metabolites in vivo. Simultaneously, these metabolites produced in vivo would be validated by fecal fermentation in vitro. Finally, the metabolic pathway of genipin could be revealed, which could establish the foundation for efficacy studies of genipin.

## 2. Results

### 2.1. Establishment of the Analytical Strategy

**Figure 1:**
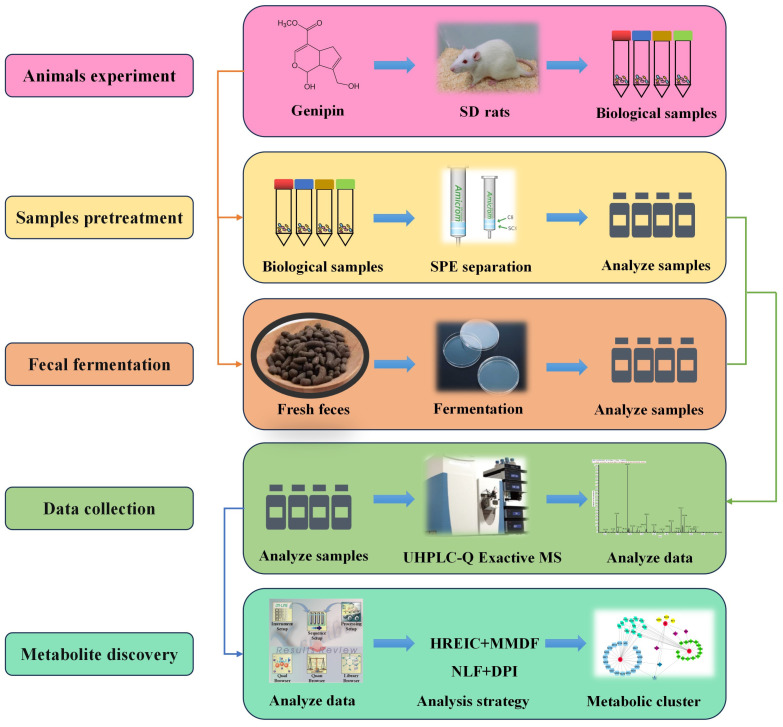
Summary of the analytical strategy and methodology.

An effective and comprehensive analytical strategy based on the UHPLC-HRMS analysis platform was established for the identification of genipin metabolites in SD rats. Firstly, the primary ESI-MS^1^ spectra were collected at a resolving power of 70,000 in negative (N) and positive (P) ion modes. Moreover, the ESI-MS^n^ datasets were obtained using full data-dependent scans. Secondly, for the subsequent post collection data mining processing, a combination of MMDF and HREIC methods were used to obtain a dataset of genipin metabolites [15]. Among them, MMDF was used to identify the MS^n^ information of unknown metabolites accurately and completely through potential metabolites and homologous substances. HREIC was utilized to monitor predictable metabolites through the common biotransformation reactions and metabolites reported in the literature [16]. Thirdly, the precise metabolites of genipin were identified based on the comprehensive analysis method of neutral fragment losses (NFLs) and characteristic ion fragments (DPIs) produced by the fragmentation reaction of ESI-MS^n^ [17]. The Clog*P* value obtained by ChemDraw was used to distinguish the metabolites’ isomers with different retention times in the ESI-MS^1^ spectra. Fourthly, based on the in vitro fecal fermentation of genipin, the metabolites in blood, urine, and feces in vivo were verified. Finally, the metabolic pathways of genipin were portrayed based on the identified metabolites and their metabolic reactions. The entire analytical strategy was shown in Figure 1.

### 2.2. Establishment of the MMDF Data-Mining Method

Multiple different MMDF templates were set for the acquisition of the unpredicted metabolites. In previous studies, genipin was converted into genipinine under the intervention of intestinal bacteria [18]. Therefore, two parallel MMDF templates were used to detect metabolites: (1) the prototype drug template (*m/z* 226.08412) and its conjugation templates in negative ion mode (*m/z* 224.06847 for dehydrogenation, *m/z* 306.04094 for sulfate conjugation); (2) genipinine (*m/z* 209.10519) and its conjugation templates in the positive ion mode (*m/z* 207.08954 for dehydrogenation, *m/z* 305.05692 for sulfate conjugation, *m/z* 193.07389 for demethyl). Moreover, some primary metabolites were also set as new templates to excavate when these metabolites were found during the subsequent identification or when the current templates did not cover the metabolic profiles of genipin.

### 2.3. DPI Based on the Mass Fragmentation Behaviors of Genipin and Genipinine

**Figure 2:**
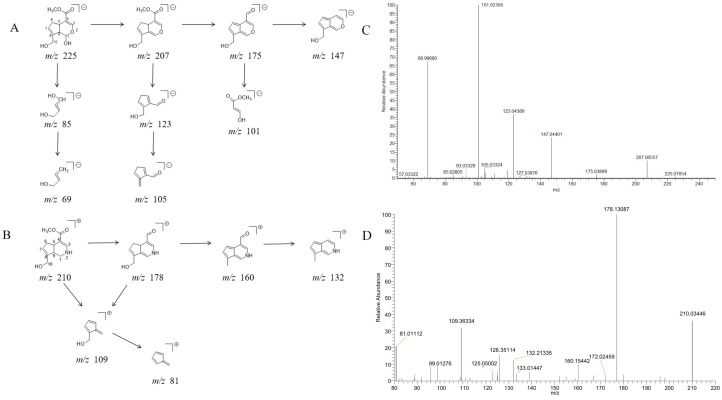
The ESI-MS/MS results of genipin and genipinine. (**A**) Genipin, (**B**) genipinine, (**C**) genipin MS^2^ spectrum, (**D**) genipinine MS^2^ spectrum.

In negative ion mode, genipin showed the [M-H]^−^ ion at *m/z* 225.07623 (C_11_H_13_O_5_, 2.133 ppm) in the ESI-MS spectrum. In the ESI-MS^2^ spectrum, it produced a series of fragment ions such as *m/z* 101 ([M-H-H_2_O-CH_4_O-C_6_H_2_]^−^), *m/z* 69 ([M-H-C_7_H_8_O_4_]^−^), *m/z* 123 ([M-H-H_2_O-C_4_H_4_O_2_]^−^), *m/z* 147 ([M-H-H_2_O-CH_4_O-CO]^−^), *m/z* 207 ([M-H-H_2_O]^−^), *m/z* 119 ([M-H-H_2_O-CH_4_O-2CO]^−^), and *m/z* 111 ([M-H-3H_2_O-CH_4_O-CO]^−^). The results are shown in Figure 2A.

Under positive ion mode, genipinine exhibited the [M + H]^+^ ion at *m/z* 210.11247 (C_11_H_14_O_4_N, −2.208 ppm) with a retention time of 7.72 min. In addition, genipinine generated the DPIs at *m/z* 178 ([M + H-CH_4_O]^+^), *m/z* 210 ([M + H]^+^), *m/z* 132 ([M + H-CH_4_O-CO-H_2_O]^+^), *m/z* 160 ([M + H-CH_4_O-H_2_O]^+^), *m/z* 109 ([M + H-C_4_H_7_NO_3_]^+^), *m/z* 81 (M + H-C_4_H_7_NO_3_-CO]^+^), and so on. The DPIs of genipinine are illustrated in Figure 2B.

### 2.4. Structural Confirmation of Genipin Metabolites

A total of 50 metabolites (prototype included) were identified in the rats’ plasma, feces, and urine based on the above analytical strategy. Among them, 28, 31, and 7 metabolites were identified in feces, urine, and plasma, respectively. It was worth noting that among all of the metabolites, 11 metabolites shared by feces and urine were found, and three metabolites shared by plasma and feces were found. The mass spectrometric data and identification results of the genipin metabolites were shown in Table 1, respectively. According to the results, it was concluded that genipin and its metabolites were mostly detected in negative ion mode, and a few of them were detected in positive ion mode. Meanwhile, the genipin metabolites were divided into two categories to be described clearly. The total ion current (TIC) figure containing each metabolite is shown in Supplementary Figure S1.

### 2.5. Identification of Genipin and Its Metabolites

**M0** was eluted at 6.38 min and exhibited the experimental [M-H]^−^ ion at *m/z* 225.07623 (C_11_H_13_O_5_, 2.133 ppm). Then, **M0** formed many same DPIs as genipin in the ESI-MS^2^ spectrum at *m/z* 101, *m/z* 69, *m/z* 123, and *m/z* 147. Meanwhile, **M0** showed the experimental ion at *m/z* 227.09140 (C_11_H_15_O_5_, −3.963 ppm) in the positive ion mode. In the MS^2^ spectra, the DPIs at *m/z* 116([M + H-C_7_H_10_O]^+^), *m/z* 209([M + H-H_2_O]^+^), *m/z* 157([M + H-C_4_H_6_O]^+^), and *m/z* 131([M + H-C_6_H_8_O]^+^) verified the agreement with the genipin fragment ions. Hence, **M0** was determined unambiguously to be genipin itself according to the reference standard. Specific results are shown in Table 1.

With a retention time of 1.03 min, **M4** afforded the [M-H]^−^ ion at *m/z* 241.07164 in negative ion mode with a mass error of 4.046 ppm. **M4** was 16 Da more than that of **M0**, and the result witnessed that **M4** might be the hydroxylation product of **M0**. In its ESI-MS^2^ spectrum, the neutral loss of 32 Da (*m/z* 241→*m/z* 209) and a few characteristic fragment ions at *m/z* 69, *m/z* 101, and *m/z* 162 confirmed that the hydroxyl group should be replaced on the five-carbon ring of genipin. Moreover, **M9** was eluted at 5.37 min, and afforded the theoretical ion at *m/z* 321.02855 (C_11_H_14_O_9_S, 3.367 ppm), which was 80 Da more than **M4**. Therefore, **M9** yielded a series of DPIs at *m/z* 321, *m/z* 241, *m/z* 101, *m/z* 139, and *m/z* 121. Among them, the neutral loss of 80 Da (*m/z* 321→*m/z* 241) revealed that **M9** might be presumed to be the sulfation product of **M4**. In addition, the DPI at *m/z* 101 indicates that the sulfonic acid group might be substituted on the hydroxyl group at position 1 of **M4**. Consequently, **M9** was preliminarily identified as the sulfation and hydroxylation product of **M0**.

**M11** was eluted at 5.63 min and generated the [M-H]^−^ ion at *m/z* 401.10895 in negative ion mode with a mass error of 3.296 ppm. Furthermore, **M11** was 176 Da more than **M0** (*m/z* 401 → *m/z* 225). Simultaneously, **M11** also generated many fragment ions at *m/z* 101, *m/z* 123, *m/z* 147, *m/z* 207, and *m/z* 69, which were consistent with those of genipin. The other fragment ions at *m/z* 325 ([M-H-C_2_H_3_O_2_-OH]^−^) and *m/z* 193 ([M-H-C_6_H_8_O_6_-CH_4_O]^−^) were observed in its ESI-MS^2^ spectrum. In summary, **M11** was tentatively identified as genipin-1-*O*-glucuronide combined with the previous literature [19].

**M6** gave rise to its [M-H]^−^ ion at *m/z* 195.06563 (C_10_H_11_O_4_, 2.280 ppm) with a retention time of 1.20 min. It was 30 Da less than that of genipin, indicating that **M6** might be a dehydroxylation and demethylation product of genipin. The appearance of the base peak ion at *m/z* 123 indicated that the hydroxyl group should be lost at the C1 position, and the other DPIs at *m/z* 177, *m/z* 119, *m/z* 133, and *m/z* 97 in the ESI-MS^2^ spectrum confirmed that the methyl group was eliminated on the ester bond at the C4 position. Therefore, **M6** was predicted to be a dehydroxylation and demethylation product of **M0**.

**M3** and **M12** were eluted at 0.99 min and 6.10 min, respectively, and possessed the same theoretical [M-H]^−^ ion at *m/z* 305.03365 (C_11_H_14_O_8_S, mass error within 5.0 ppm) in negative ion mode, which was 80 Da more than that of genipin. At the same time, they produced the base peak ion at *m/z* 101 and a series of fragment ions at *m/z* 123, *m/z* 69, *m/z* 147, and *m/z* 207, which also appeared in the spectrum of genipin. In addition, **M3** and **M12** also generated a fragment ion at *m/z* 225 ([M-H-SO_3_]^−^). In summary, **M3** and **M12** were tentatively identified as sulfation products of genipin.

**M21** and **M28** were eluted at 6.82 min and 7.39 min, respectively, and yielded the same theoretical ion at *m/z* 197.08195 (C_11_H_14_O_4_, mass error within 3.0 ppm). They were 28 Da less than that of genipin in negative ion mode. In the ESI-MS^2^ mass spectra, **M21** and **M28** both generated the base peak ion at *m/z* 153 due to the neutral loss of 44 Da (-CO_2_), and the multiple DPIs at *m/z* 197 ([M-H]^−^), *m/z* 179 ([M-H-H_2_O]^−^), and *m/z* 135 ([M-H-H_2_O-CO_2_]^−^) revealed that **M21** and **M28** might be decarbonylation products of genipin. Finally, combined with the other DPIs at *m/z* 69, *m/z* 123, and *m/z* 59, **M21** and **M28** could be identified as decarbonylation products of genipin.

**M40** and **M49**, which were 14 Da higher than that of genipin, showed their theoretical [M-H]^−^ ion at *m/z* 239.09255 (C_12_H_16_O_5_, mass error within 5.0 ppm). **M40** and **M49** both generated multiple DPIs at *m/z* 239 ([M-H]^−^), *m/z* 221 ([M-H-H_2_O]^−^), *m/z* 195 ([M-H-CO_2_]^−^), *m/z* 177 ([M-H-CO_2_-H_2_O]^−^), and *m/z* 151 ([M-H-CO_2_-H_2_O-CO]^−^). In addition, the neutral loss of 116 Da (*m/z* 239 → *m/z* 123) and 88 Da (*m/z* 239 → *m/z* 151) confirmed that **M40** and **M49** were preliminarily identified as 3-methyl-genipin or its isomer.

**M32** was eluted at 7.76 min with a mass error of 0.515 ppm (C_10_H_16_O_4_), which yielded the [M-H]^−^ ion at *m/z* 199.09432 in negative ion mode. At the same time, **M32** was 26 Da less than genipin, and it could generate DPIs at *m/z* 155 ([M-H-CO_2_]^−^), *m/z* 199 ([M-H]^−^), *m/z* 181 ([M-H-H_2_O]^−^), and *m/z* 137 ([M-H-CO_2_-H_2_O]^−^). This fragmentation behavior revealed that **M32** might contain a carbonyl group and hydroxyl group. Meanwhile, the DPIs at *m/z* 130 and *m/z* 59 indicated that **M32** could be a ring-opening product of the oxygen-containing heterocycle in the genipin structure.

**M17** showed the theoretical [M-H]^−^ ion at *m/z* 209.04549 (C_10_H_10_O_5_, mass error of 2.536 ppm), which was eluted at a retention time of 6.45 min. Moreover, it was 16 Da less than that of genipin. The base peak ion at *m/z* 121 due to the neutral loss of 88 Da (−2CO_2_) and the fragment ions at *m/z* 181 ([M-H-CO]^−^) and *m/z* 69 (loss of 140 Da) revealed that **M17** might be the demethylation product of genipin after the dehydrogenation reaction, and then the hydroxyl group should on positive 1 because of the DPI at *m/z* 136. **M15** was 16 Da less than **M17**, which was eluted at 6.29 min and showed the [M-H]^−^ ion at *m/z* 193.05069 (C_10_H_10_O_4_) with a mass error of 1.941 ppm. The base peak ion at *m/z* 133 ([M-H-CO_2_-O]^−^) was detected in the ESI-MS^2^ mass spectrum. Furthermore, the DPIs at *m/z* 178 ([M-H-CH_3_]^−^) and *m/z* 149 ([M-H-CO_2_]^−^) confirmed that **M15** was presumed to be the demethylation product of **M17**.

**M35**, **M37**, and **M39** afforded the same theoretical ion at *m/z* 211.09819 (C_11_H_16_O_4_, mass error within 4.00 ppm), which were eluted at 8.04 min, 8.18 min, and 8.32 min, respectively. Among them, they were 14 Da less than the [M-H]^−^ ion at *m/z* 225.07623 of genipin, and all generated a series of DPIs at *m/z* 167 ([M-H-CO_2_]^−^), *m/z* 149 ([M-H-CO_2_-H_2_O]^−^), and *m/z* 132 ([M-H-CO_2_-H_2_O]^−^). Therefore, **M35**, **M37**, and **M39** were initially identified as dehydroxylation and hydrogenation products of genipin or its isomers. Moreover, the fragment ions at *m/z* 99 and *m/z* 59 confirmed that two hydrogens were added to the pyran ring of genipin and the hydroxyl group was lost at position 8. Thus, **M35**, **M37**, and **M39** could be identified as dehydroxylation and hydrogenation products of genipin or its isomers. **M23** was 16 Da less than **M35**, **M37**, and **M39**, which was eluted at 6.89 min and yielded the [M-H]^−^ ion at *m/z* 195.10265 (mass error of 2.251 ppm), demonstrating that it might be a dehydroxylation product of **M35**, **M37**, and **M39**. Simultaneously, the DPIs at *m/z* 151 ([M-H-CO_2_]^−^), *m/z* 177 ([M-H-H_2_O]^−^), *m/z* 165 ([M-H-CH_2_O]^−^), and *m/z* 149 ([M-H-CO-H_2_O]^−^) also confirmed the above statement. Consequently, **M23** could be identified as a dehydroxylation product of **M35**, **M37**, and **M39**, in which the hydroxyl group was lost at position 1 based on the two DPIs at *m/z* 80 and *m/z* 165.

**M38** yielded the [M-H]^−^ ion at *m/z* 179.10775 with the mass error of −0.234 ppm, and thus, the molecular formula was presumed to be C_11_H_16_O_2_. **M38** was 16 Da less than **M23**. In its ESI-MS^2^ mass spectrum, the DPIs at *m/z* 179 ([M-H]^−^), *m/z* 161 ([M-H-H_2_O]^−^), and *m/z* 135 ([M-H-H_2_O-CO]^−^) confirmed **M38** should be a deoxygenation and demethylation product of **M23**. Moreover, the special DPI at *m/z* 59 (RDA rearrangement occurs on the six-membered ring) also revealed that the oxygen atom was abandoned from the six-membered heterocycle. The DPI at *m/z* 80 also confirmed that there was a carboxyl group in the molecule of **M38**. Thus, **M38** could be identified as a deoxygenation and demethylation product of **M23**.

**M41**, **M43**, **M44**, and **M45,** with the same theoretical [M-H]^−^ ion at *m/z* 213.11270 (C_11_H_18_O_4_, mass error within 4.00 ppm), were eluted at 8.32 min, 8.43 min, 8.53 min, and 8.69 min, respectively, and were 2 Da higher than that of **M35**, **M37**, and **M39**. Based on the behavior of DPIs at *m/z* 169, *m/z* 213, *m/z* 151, and *m/z* 195, **M41**, **M43**, **M44**, and **M45** were identified as the hydrogenation products of **M35**, **M37**, and **M39**. Their structures were tentatively elucidated in Table 1. **M25** and **M29** were 176 Da higher than **M41**, **M43**, **M44**, and **M45**, which were eluted at 7.35 min and 7.43 min, respectively. They showed the same theoretical ions at *m/z* 389.14480 with the mass errors of 3.229 ppm and 2.818 ppm. In the ESI-MS/MS spectra, the neutral loss of 176 Da (*m/z* 389 → *m/z* 213) indicated the presence of the glucuronidation group in the structure of **M25** and **M29**. Therefore, **M25** and **M29** were identified as glucuronidation products of **M41**, **M43**, **M44**, and **M45**. Meanwhile, the ions at *m/z* 80 and other mass fragment behaviors of *m/z* 213, *m/z* 235, and *m/z* 59 revealed that the glucuronidation group was only added into position 1 of **M41**, **M43**, **M44**, and **M45**.

**M34** afforded the [M-H]^−^ ion at *m/z* 223.06115 (C_11_H_12_O_5_) with the mass error of 2.690 min, which was eluted at 8.00 min. Meanwhile, **M34** was 2 Da less than genipin, indicating that **M34** was the dehydrogenation product of genipin. Meanwhile, the DPIs at *m/z* 223, *m/z* 179, *m/z* 161, and *m/z* 195 in its ESI-MS^2^ mass spectrum confirmed the above deduction. In addition, other DPIs at *m/z* 143 and *m/z* 155 further indicated that two hydrogens should be removed in the junction of the furan ring and the oxygen-containing heterocyclic ring. Therefore, **M34** was tentatively identified as the dehydrogenation product of genipin, and its structure is displayed in Table 1. **M46**, which was eluted at 8.82 min, afforded the [M + H]^+^ ion at *m/z* 209.08087 (C_11_H_12_O_4_) with the mass error of −1.269 ppm in positive ion mode. At the same time, **M46** was 16 Da less than that of **M34**, providing evidence that **M46** was the dehydroxylated product of **M34**. The fragment ion at *m/z* 121, which was generated due to the neutral loss of 88 Da (2CH_2_O + CO), could not only provide evidence for the above speculation, but also illustrated that the hydroxyl group was probably abandoned from the oxygen-containing heterocycle. Multiple DPIs at *m/z* 149, *m/z* 177, *m/z* 209, and *m/z* 131 identically supported the above inference. Therefore, **M46** was initially identified as the dehydroxylated product of **M34**.

**M48** was eluted at 9.45 min and afforded the [M-H]^−^ ion at *m/z* 359.09839 with the mass error of −0.193 ppm. The neutral loss of 176 Da (*m/z* 359 → *m/z* 183) validated the presence of glucuronic acid, and the base peak ion at *m/z* 297 was generated due to the loss of 62 Da (CO_2_ + H_2_O). The DPIs at *m/z* 171, *m/z* 143, *m/z* 125, and *m/z* 82 confirmed the existence and substitution position of the glucuronic acid group. Thus, **M48** was identified as a glucuronic acid product.

### 2.6. Identification of Genipinine and Its Metabolites

**M31** was eluted at 7.72 min yielded the theoretical ion at *m/z* 210.11247 (C_11_H_15_O_3_N, mass error of −1.457 ppm) in positive ion mode. The fragmentation behavior of **M31** was exactly the same as that of genipinine. Thus, **M31** was inferred to be genipinine. Specific results are shown in Table 2.

**M1** was eluted at 0.81 min and afforded the [M + H]^+^ ion at *m/z* 120.06567 (C_4_H_9_O_3_N, mass error of −0.247). **M1** generated DPIs at *m/z* 120 ([M + H]^+^), *m/z* 103 ([M + H-OH]^+^), *m/z* 91 ([M + H-CH_5_N]^+^), and *m/z* 106 ([M + H-CH_2_]^+^). Therefore, **M1** could be identified as 3-hydroxy-3-(methylamino)propanoic acid. **M2** was eluted at 0.82 min with the mass error of −2.205 ppm, and its molecular formula could be deduced as C_7_H_7_O_3_N. The DPIs at *m/z* 154, *m/z* 136, and *m/z* 110 indicated that **M2** was 5-(hydroxymethyl) nicotinic acid.

**M19** was eluted at 6.56 min, which was 56 Da more than genipinine, indicating that it could be the glycine conjugation product of genipinine (C_13_H_18_O_4_N_2,_ −2.035 ppm). In addition, the base peak ion at *m/z* 134 was generated due to the neutral loss of 133 Da, and other DPIs at *m/z* 239 (CO), *m/z* 161 (2CO + CH_4_O + H_2_O), and *m/z* 207 (CO + CH_4_O) revealed the above deduction. The esterification reaction occurred between the glycine group and the hydroxyl group at position 10 of genipinine. Consequently, **M19** was identified as the glycine-binding metabolite of genipinine.

**M20** (C_11_H_13_O_4_N, mass error of −2.208) was 14 Da more than genipinine, which was eluted at 6.66 min, indicating that the carbonyl group was introduced into its structure instead of the methyl group. In addition, the DPIs at *m/z* 224, *m/z* 206, and *m/z* 178 also confirmed the above inference; other DPIs at *m/z* 146, *m/z* 192, *m/z* 118, and *m/z* 82 further indicated that the carbonyl group could be introduced into the nitrogen-containing heterocycle of genipinine. Thus, **M20** was identified as the carbonylation product of genipinine.

**M22** was eluted at 6.88 min with the [M + H]^+^ ion at *m/z* 212.09167 (C_10_H_13_O_4_N, −1.954 ppm). The DPIs at *m/z* 109 (neutral loss of 133 Da) and *m/z* 166 ([M + H-COOH]^+^) indicated that the methyl group should be lost and the hydroxyl group was introduced. Another DPI at *m/z* 81 also revealed that the hydroxyl group should be introduced into the nitrogen-containing heterocycle. Furthermore, **M22** was preliminarily identified as the demethylation and hydroxylation product of genipinine.

**M27**, which yielded the [M + H]^+^ ion at *m/z* 306.06417 (C_11_H_16_O_7_NS) with the mass error of −0.062 ppm, was eluted at 7.36 min. Moreover, **M27** was 96 Da more than that of genipinine, indicating that **M27** could be deduced as a sulfation product of genipinine; the neutral loss of 96 Da (*m/z* 306 → *m/z* 210) confirmed the above assumption. Thus, **M27** was identified as a sulfation product of genipinine.

**M47**, which afforded the theoretical [M + H]^+^ ion at *m/z* 356.13402 with the retention time of 9.40 min (mass error of −0.508 ppm), was 146 Da more than that of genipinine in positive ion mode. The base peak ion at *m/z* 135 was formed due to the loss of 221 Da, indicating that **M47** might contain a glucuronic acid group. In addition, the neutral loss of 176 Da (*m/z* 356 → *m/z* 180) proved the presence of glucuronic acid. Combined with the DPIs at *m/z* 93 and *m/z* 107, **M47** was identified as the dehydroxylation and glucuronidation product of genipinine.

**M13** yielded the [M + H]^+^ ion at *m/z* 208.09682, which was eluted at 6.12 min with the mass error of −2.210 ppm. **M13** was 2 Da less than genipinine, and the fragment ions at *m/z* 208, *m/z* 162, and *m/z* 120 jointly indicated that **M13** could be a dehydrogenation product of genipinine. Furthermore, another DPI at *m/z* 166 also supported this evidence. Therefore, **M13** was initially identified as the dehydrogenation product of genipinine. **M18** and **M24** were eluted at 6.49 min and 7.00 min, respectively, with the same [M + H]^+^ ion at *m/z* 206.08107 (C_11_H_11_O_3_N, mass error within 1.00 ppm). In addition, **M18** and **M24** were 2 Da less than that of **M13**, so **M18** and **M24** could be identified as the dehydrogenation products of **M13**. The DPIs at *m/z* 188 and *m/z* 170 were generated due to the loss of 18 Da (H_2_O) and 36 Da (2H_2_O). The fragment ions at *m/z* 118, *m/z* 130, and *m./z* 146 could also prove the above inference. Moreover, the DPIs at *m/z* 116 indicated that two hydrogens should be dispelled on the nitrogen heterocycle of **M13**. Similar to **M18** and **M24**, **M8** also afforded the [M + H]^+^ ion at *m/z* 206.08107 (the same molecular formula, C_11_H_11_O_3_N), which was 2 Da less than **M13**, indicating that **M8** was also a dehydrogenation product of **M13**. However, **M8** could be eluted at 5.33 min. A series of DPIs at *m/z* 146, *m/z* 174, *m/z* 178, *m/z* 156, and *m/z* 91 indicated that **M8** could be identified as the dehydrogenation product of **M13**. Furthermore, **M8**, **M18**, and **M24** were isomers. No more than, because of the dehydrogenation reaction, the structure of **M8** was different from that of **M18** and **M24**. **M5** and **M33** were 16 Da more than that of **M18** and **M24** (C_11_H_11_O_4_N, mass error ≤ 1.00 ppm), respectively. Moreover, they could generate the same DPIs at *m/z* 222 ([M + H]^+^), *m/z* 204 ([M + H-H_2_O]^+^), *m/z* 162 ([M + H-CH_4_O-CO]^+^), *m/z* 144 ([M + H-CH_4_O-CO-H_2_O]^+^), and so on. Thus, **M5** and **M33** were identified as the hydroxylated products of **M18** and **M24**. Merely, according to the ESI-MS^2^ mass spectra, the position of the hydroxyl group could not be determined according to the known data. **M42** yielded the theoretical [M + H]^+^ ion at *m/z* 190.08632 with the mass error of −0.365 ppm in the positive ion mode, and its molecular formula was C_11_H_11_O_2_N. In the ESI-MS^2^ mass spectrum, **M42** could generate DPIs at *m/z* 172, *m/z* 162, and *m/z* 130 due to the neutral loss of 18 Da (H_2_O), 28 Da (CO), and 60 Da (CH_4_O + CO). Thus, **M42** could be identified as a dehydroxylated product of **M18** and **M24**. At the same time, the DPI at *m/z* 55 indicated the position of the hydroxyl group, and the possible structure of **M42** is shown in Table 1. **M26** was 2 Da less than **M18** and **M24** in the positive ion mode, which was eluted at 7.35 min with the mass error of 3.229 ppm. The base peak ion at *m/z* 204 demonstrated that **M26** should be the dehydrogenation product of **M18** and **M24**. The DPIs at *m/z* 175 and *m/z* 144 reflected the real situation of the dehydrogenation reaction, and the special DPIs at *m/z* 116, *m/z* 92, and *m/z* 65 indicated the position where the dehydrogenation reaction might occur. Thus, **M26** could be identified as the dehydrogenation product of **M18** and **M24**.

**M7** and **M10** were eluted at 4.86 min and 5.48 min, respectively. They possessed the same theoretical [M + H]^+^ ion at *m/z* 194.08112 (C_10_H_11_O_3_N, mass error within 1.00 ppm). They were 16 Da less than the DPI at *m/z* 210 in the ESI-MS^2^ mass spectrum of genipinine. The results indicated that **M7** and **M10** could be the demethylation and dehydrogenation products of genipinine, and the DPIs at *m/z* 91, *m/z* 76, *m/z* 57, *m/z* 108, and *m/z* 67 not only supported the above evidence, but also noted the positions where the methyl group and two hydrogens were possibly lost. Their inferred structures are shown in Table 1.

**M14**, which was eluted at 6.20 min, provided the fragment [M + H]^+^ ion at *m/z* 198.07617 (C_9_H_11_O_4_N, mass error of −1.537 ppm). In addition, the base peak ion at *m/z* 138 could be generated due to the neutral loss of 60 Da (CH_3_OH + CO). Moreover, other DPIs at *m/z* 130 ([M + H-CH_3_OH-2H_2_O]^+^), *m/z* 110 ([M + H-CH_3_OH-2CO]^+^), *m/z* 123 ([M + H-CH_3_OH-CO_2_]^+^), and *m/z* 108 ([M + H-CO_2_-CO-H_2_O]^+^) indicated the presence of hydroxyl groups and carbonyl groups in the **M14** structure. Thus, the **M14** structure is shown in Table 1. **M36** was 4 Da higher than genipinine, which was eluted at 8.06 min (C_11_H_19_O_3_N, mass error of −2.382 ppm). The DPI at *m/z* 214 revealed that **M36** could be the hydrogenation product of genipinine. Other DPIs at *m/z* 70, *m/z* 116, *m/z* 71, and *m/z* 168 also supported the above evidence. Furthermore, **M14** was preliminarily identified as the hydrogenation product of genipinine.

### 2.7. Identification of M16 and M30

**M16** could afford the [M + H]^+^ ion at *m/z* 226.10738 (C_11_H_15_O_4_N, mass error of −1.745 ppm) in the positive ion mode, which was eluted at 6.43 min. At the same time, the neutral loss of 32 Da (*m/z* 226 → *m/z* 194) indicated the presence of CH_3_O. The DPI at *m/z* 180 could be generated due to the loss of 46 Da (H_2_O + CO). Other DPIs at *m/z* 151, *m/z* 120, and *m/z* 148 revealed the possible structure of **M16**, which is shown in Table 1.

**M30** was eluted at 7.45 min and yielded the theoretical ion at *m/z* 225.07685 (C_11_H_14_O_5,_ mass error of 2.977 ppm), whose molecular formula was exactly same as that of genipin. However, in the ESI-MS^2^ mass spectrum, the fragmentation ions at *m/z* 163 ([M-H-CO_2_-H_2_O]^−^), *m/z* 207 ([M-H-H_2_O]^−^), and *m/z* 181 ([M-H-H_2_O-CO]^−^) indicated that **M30** was not genipin but its isomer. The DPI at *m/z* 137 ([M-H-2CO_2_]^−^) speculated that the structure of **M30** contained two aldehyde groups. Therefore, **M30** was initially identified as the aldehyde product of genipin after the partial ring opening of hydroxy acetone.

### 2.8. Identification of Genipin In Vitro Fecal Fermentation and Its Metabolites

Based on the distributional characteristics of genipin metabolites, we verified these metabolite results by fermenting genipin with rat feces in vitro. Forty-six metabolites were discovered in the fecal fermentation system. Among them, metabolite **M0** (genipin) could be found at 0 h in vitro. **M1** and **M4** could all be analyzed from 0 h to 60 h of fermentation, which had never been found in control fermentations (excluding genipin). Therefore, **M1** and **M4** were inferred to be the primary metabolites of **M0**. Subsequently, most of the metabolites were found at 12 h of fecal fermentation in vitro, indicating that genipin could be rapidly metabolized to produce secondary metabolites. Simultaneously, we found that the previously identified metabolites were consistently discovered during 24 h to 36 h of fermentation in vitro. **M38** could be detected at 24 h, 48 h, and 60 h without 36 h, suggesting that **M38** might initially be generated at 24 h, and be degraded to other metabolites at 36 h, and then again formed by other metabolites at 48 h. In addition, metabolites **M27**, **M47**, and **M48** could be also found at 48 h; only **M48** was continued to be identified at 60 h of fermentation in vitro, while the undiscovered results of **M27** and **M47** at 60 h indicated that they should be degraded into other metabolites. Meanwhile, metabolites **M4**, **M5**, **M6**, **M14**, **M15**, **M16**, **M21**, **M27**, **M31**, **M33**, **M40**, and **M47** could not be detected at 60 h in vitro fermentation, suggesting the formidable degradation of intestinal bacteria. Combined with the metabolite results in vitro and in vivo, we found that 21 metabolites identified in blood and urine could also be detected during different times in vitro fecal fermentation such as **M1**, **M22**, **M5**, **M14**, **M18**, **M19**, **M20**, and **M24**. Among them, **M1** and **M22** were analyzed in blood and 19 metabolites were discovered in urine such as **M37**, **M39**, **M40**, **M47**, and **M49**. These results suggested that these metabolites identified in blood and urine might be first produced in feces and then reabsorbed into the body such as **M27**, **M27**, **M29**, and **M33**, which is just a hypothesis.

## 3. Discussion

In the study of drug metabolism, the structural identification of metabolites is one of the most important tasks [20,21,22]. Therefore, a new analytical strategy based on UHPC-HRMS combined with multiple data processing methods was applied to effectively identify the metabolites of genipin in rats. In the traditional mode, metabolic profiling is displayed in the unitary mode, which may cause the loss of multiple metabolites [23,24,25]. In this study, positive and negative ion modes, combined with other data analysis methods, were applied to complete the comprehensive analysis of genipin metabolites. As a result, a total of 50 metabolites were identified, most of which were identified and reported for the first time. Among them, a total of 30 metabolites were identified with genipin as the metabolic center involving genipin (**M0**), sulfation (**M3**), hydroxylation (**M4**), dehydroxylation and demethylation (**M6**), sulfation and hydroxylation (**M9**), glucuronidation (**M11**), sulfation (**M12**), demethylation (**M17**), decarbonylated product (**M21**), glucuronidation (**M25**, **M29**), dehydroxylation (**M46**), and so on. Meanwhile, a total of 20 metabolites were identified with genipinine as the metabolic center containing debenzylation (**M1**), hydroxylated products (**M5**, **M33**), demethylated and dehydrogenated product (**M7**), dehydrogenation (**M8**), dehydrogenated product (**M18**), demethylation and hydroxylation (**M22**), sulfated product (**M27**), genipinine (**M31**), hydrogenation (**M36**), dehydroxylation (**M42**), dehydroxylation and glucuronidation(**M47**), and so on. Furthermore, it is worth noting that the majority of all metabolites were from feces. This suggests that the metabolites of genipin have better recognition ability in feces.

**Figure 3:**
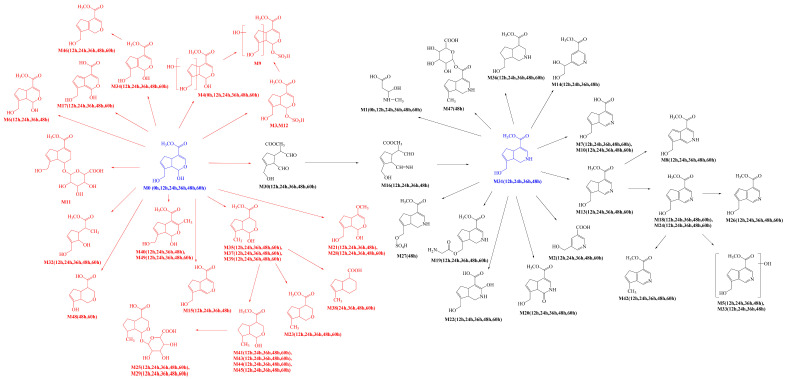
The proposed metabolic pattern of genipin in vivo and vitro.

In the process of drug metabolism, the parent drug usually forms intermediate metabolites, which are also known as one-phase metabolites. Then, intermediate metabolites can be metabolized into two-phase metabolites by many further metabolic reactions. Based on this phenomenon, we used “metabolite clusters” to express the relationship between genipin and its metabolites (Figure 3). The centers of the metabolic clusters were genipin and genipinine. In addition, there were two metabolites (**M16** and **M30**) in the metabolic pathway of genipin to genipinine, and the results showed the existence of the transformation pathway in four substances, which has been reported in previous literature [26]. Moreover, many studies have noted that sulfation products are the main component of genipin metabolites, and metabolites **M3**, **M9**, **M12**, and **M27** also confirmed the above statement [27,28,29].

In order to fully validate the metabolic results in vivo, a fecal fermentation in vitro was used to verify the metabolites of genipin in vivo. Therefore, a total of 46 metabolites were identified in vitro fecal fermentation. Most of the metabolites in urine and feces in vivo were identified in vitro, suggesting that the environment of fecal fermentation in vitro was compatible with that in vivo. Twelve metabolites could appear at 48 h, and were not detected at 60 h. The result suggests that other metabolic reactions occurred rapidly after genipin conversion, and then degraded to participate in the physiological function. Furthermore, the number of metabolites peaked at 12 h of fecal fermentation in vitro, indicating that genipin could be rapidly transformed into the related metabolites within 12 h. Notably, two metabolites in blood and 19 metabolites in urine such as **M1**, **M22**, **M5**, and **M14** were still detected in fecal fermentation, suggesting that these metabolites might be absorbed into the circulatory system after fecal metabolism. Finally, **M31** was detected as early as 12 h in vitro fecal fermentation, and in the previous analytical strategy, genipin was converted to the genipinine base in vivo under the action of gut microbiota, and this beneficial effect was demonstrated in in vitro fecal fermentation. The detailed distribution results of the metabolites and fecal fermentation at different times are illustrated in Supplementary Table S1.

## 4. Materials and Methods

### 4.1. Chemicals and Materials

The reference standard such as genipin (batch number: MUST-11052312) was purchased from Chengdu Must Biotechnology Co. Ltd. (Sichuan, China). Its structure has been perfectly elucidated by comparing the spectral data (ESI-MS^1^, ^1^H-NMR) with the published literature. The purity of genipin was acceptable (≥99.8%) according to HPLC-UV analysis. Anaerobic gas producing bag, sterile Petri dish, anaerobic culture bag, Gifu anaerobic medium (GAM), vitamin K1, and hemin chloride were purchased from Qingdao Haibo Biotechnology Co. Ltd. (Shandong, China). Oxygen indicators were purchased from Mitsubishi Corporation, Japan.

The LC-MS grade acetonitrile, methanol, and formic acid (FA) were purchased from Thermo Fisher Scientific (Fair Lawn, NJ, USA). Deionized water was purchased from Watsons Group Ltd. (Hong Kong, China). Grace pure™ SPE C18 solid-phase extraction cartridges (300 mg/3 mL, 5.0 μm, 60 Å) were purchased from Grace Davison Discovery Science (Deerfield, USA).

### 4.2. Identification of Genipin Metabolites In Vivo

#### 4.2.1. Animals

Eight male SD rats (200 g ± 20 g) were obtained from Jinan Pengyue Experimental Animal Breeding Co. Ltd. (Jinan, China, raised in a regulated environment (temperature of 24 ± 2 °C, humidity of 70 ± 5%)m, and kept on a 12 h light/12 h dark alternating cycle. After 7 days of adaptive feeding, all rats were divided into two groups: the control group (*n* = 4) for control plasma, urine, and feces and the drug group (*n* = 4) for testing urine, blood, and feces. They were fasted for 12 h with free access to water prior to the experiment. The animal experiment protocols were approved by the Animal Care and Use Committee of Binzhou Medical University.

#### 4.2.2. Drug Administration and Biological Sample Preparation

Drug metabolism in vivo is a complex biological transformation process, and the metabolites produced in the animal itself will interfere with the subsequent drug metabolism research. Therefore, control plasma, urine, and feces were set up to distinguish the metabolites unique to genipin. Genipin was suspended in 0.5% carboxymethylcellulose sodium (CMC-Na) solution. All of the rats in the drug group were given a dose of 250 mg/kg [30,31,32]. Meanwhile, 2.0 mL of CMC-Na solution was given to rats in the blank group. Blood samples were collected from the ocular venous plexus of rats from two groups at 0.5, 1, 1.5, 4, and 6 h post-administration [33]. In addition, all of the blood samples were centrifuged at 3500 r/min for 15.0 min to obtain plasma. After 3 days of continuous intragastric administration, SD rats were fasted and 24 h of urine and feces were collected from each SD rat. Moreover, all of the feces samples (1.0 g) were dissolved into 5.0 mL methanol and was extracted by an ultrasonic extraction instrument at 50 HZ for 30.0 min at room temperature. Next, the extracts of all of the feces samples were collected after centrifugation at 4500 rpm for 10.0 min at 4 °C. Finally, all samples of the rat’s blood, urine, and feces from the same group were assigned to a collective sample [34].

Proteins and solid residues in the biological samples could interfere with the metabolite signals in ESI-MS/MS analysis [35]. Therefore, an effective method involving protein and solid residue precipitation and concentration was applied to all of the biological samples [36]. The samples including blood, urine (1.0 mL), and feces were respectively added to the SPE columns, which were preliminarily activated with 5.0 mL of methanol and 5.0 mL of deionized water. Subsequently, the SPE cartridges were flushed with 3.0 mL of deionized water and 3.0 mL of methanol. Finally, the methanol eluate was collected and then evaporated using a nitrogen blowing instrument at room temperature. The remains were reconstituted using a 100.0 μL mixed solution of acetonitrile and water (90:10, *v*/*v*).

### 4.3. Biotransformation In Vitro of Genipin by SD Rat Feces

Fresh feces from SD rats in the control group were collected on the day of the experiment. A total of 5.5 g of fresh feces was added to saline at a 1:9 ratio and immediately mashed with a tissue homogenizer. The supernatant was collected after centrifugation of the fecal suspension at 4500 rpm. Then, 49.0 g of GAM broth was dissolved in 1000 mL water and sterilized under high pressure (0.15 MPa) and temperature (121 °C) for 15.0 min. A total of 1.0 mg vitamin K1 and 5.0 mg hematin chloride were dissolved in the above solution after being cooled under aseptic conditions. The final ratios of fecal supernatant and GAM broth were as follows: 50% fecal slurry, 50% GAM broth. Genipin was separately added into the mixture to make a 0.5 mg/mL stock solution. Meanwhile, 4.0 mL of the stock solution was added to different Petri dishes, and a negative control group without genipin and a control GAM broth group were set up. The mixture was incubated in anaerobic bags with an oxygen indicator (MGC, Tokyo, Japan) at 37 °C, and the samples were collected at 0 h, 12 h, 24 h, 36 h, 48 h, and 60 h, respectively. All studies were repeated three times to obtain accurate results. Each parallel sample was mixed, and 1.0 mL of the mixed sample was vortexed and mixed by adding cold methanol at 1:4. It was placed in the refrigerator at 4 °C for 12 h to remove the impurities. After centrifugation at 12,000 rpm for 5.0 min, the supernatant was taken for analysis.

### 4.4. Instrument and Conditions

LC analysis was performed on a DIONEX Ultimate 300 UHPLC system (Thermo Fisher Scientific, MA, USA) with a binary pump, an autosampler, and a column oven. Chromatographic separation of all samples was carried out using an Ultimate AQ-C18 column (2.1 × 100 mm, 1.7 µm, 60Å, Thermo Fisher Scientific, Waltham, MA, USA) at a column temperature of 40 °C. Acetonitrile (phase B) and 0.1% FA aqueous solution (phase A) were used as the mobile phases with the linear gradient elution set as follows: 0–2.0 min, 5% B; 2.0–4.0 min, 5–40% B; 4.0–9.0 min, 40–60% B; 9.0–16.0 min, 60–5% B; 16.0–20.0 min, 5% B. The flow rate was 0.35 mL/min.

ESI-MS/MS analysis was performed using a Q-Exactive Orbitrap mass spectrometer (Thermo Fisher Scientific, MA, USA). The specific parameters in negative ion mode were set as follows: sheath gas flow rate of 35 arb, spray voltage of 4500 V, auxiliary gas flow rate of 25 arb, capillary voltage of −35 V, capillary temperature of 350 °C, and tube lens of −110 V. Furthermore, in positive ion mode, the related parameters were set as follows: sheath gas flow rate of 30 arb, spray voltage of 4000 V, auxiliary gas flow rate of 35 arb, capillary voltage of −35 V, capillary temperature of 325 °C, and tube lens of 110 V. The metabolites were detected using full-scan MS analysis from *m/z* 70–1050 at a resolving power of 70,000 FWHM. The collision energy of collision-induced dissociation (CID) was set to 35%.

## 5. Conclusions

In this study, a more complete metabolic analytical strategy was established and the in vivo metabolites were validated by in vitro fecal fermentation at different times. Compared with the previously reported analysis and identification methods, UHPLC-HRMS technology combined with metabolic clusters has various advantages. On the one hand, UHPLC-HRMS can comprehensively detect and provide high-quality mass spectrometry data and multistage mass spectrum datasets. On the other hand, the method of metabolic clusters could be a more logical and systematic identification technique that could guide metabolite classification, metabolic process interpretation, and metabolic pathway enrichment. In vitro fecal fermentation experiments demonstrated that different metabolites were produced at different times in the body, and the metabolites produced in the feces were reabsorbed into the body for biotransformation. This provides a good reference value for the further study of genipin. Unfortunately, this study has some limitations such as the lack of the quantitative analysis of genipin during in vitro fecal fermentation to infer the rate at which metabolic reactions proceed at different time points. In summary, the quantitative relationship between genipin and fecal fermentation will be further revealed in future studies.

## Figures and Tables

**Table 1:** Summary of genipin as the metabolic center detected in vivo metabolites and in vitro fecal fermentation at different times.

Peak	Product	t_R_ /min	Formula	Theoretical Mass (*m/z*)	Experimental Mass (*m/z*)	Error (ppm)	MS/MS Fragment Ions	Identification	Vivo			Vitro
									U	B	F	Different Time (h)
**M0**	Genipin	6.38	C_11_H_13_O_5_	225.07685	225.07623	2.133	**101(100)**,** 69(68)**, **123(37)**, **147(24)**, **207(10)**, **119(4.5)**, **111(2.4)**	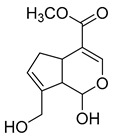			N	0 h(N), 12 h(N), 24 h(N), 36 h(N), 48 h(N), 60 h(N)
		6.38	C_11_H_15_O_5_	227.09140	227.09050	−3.963	**116(100)**, 70(92), **209(23), 157(5), 131(4)**, 85(3)					0 h(P), 12 h(P), 24 h(P), 36 h(P), 48 h(P), 60 h(P)
**M3**	Sulfation	0.99	C_11_H_13_O_8_S	305.03365	305.03381	4.085	**101(100)**, **123(64)**, **69(39)**, **305(27)**, **225(16)**, **147(15)**, 97(14), **81(13)**,**207(12)**, **273(9.3)**, **111(8.7)**	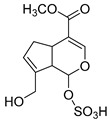	N		N	
**M4**	Hydroxylation	1.03	C_11_H_13_O_6_	241.07121	241.07164	4.046	**69(100)**, 165(53),** 209(33)**, **162(29), 101(12)**, **241(10)**, 80(6.3)	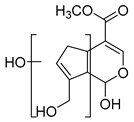		N	N	0 h(N), 12 h(N), 24 h(N), 36 h(N), 48 h(N), 60 h(N)
**M6**	Dehydroxylation and demethylation	1.20	C_10_H_11_O_4_	195.06629	195.06563	2.280	**123(100)**, **133(51)**, 81(43), 135(32), **177(30)**, **59(21)**, **97(17)**, **119(15)**	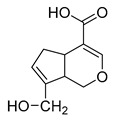			N	12 h(N), 24 h(N), 36 h(N), 48 h(N)
**M9**	Sulfation and hydroxylation	5.37	C_11_H_13_O_9_S	321.02855	321.02856	3.367	**101(100)**, 97(81), 127(74), 109(55), **321(45)**, 67(28),**139(20)**, 79(18), **241(16)**, 81(15), **209(12)**, **111(12)**,**121(10)**	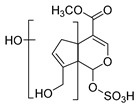	N		N	
**M11**	Genipin-1-*O*-glucuronide	5.63	C_17_H_20_O_11_	401.10895	401.10883	2.474	**101(100)**, **123(67)**, **401(63**), **69(57)**, 113(48), **85(28)**, **207(18)**, **147(16)**, 369(13), **105(11)**, 87(11), **325(10)**, **193(10)**, **225(2.3)**	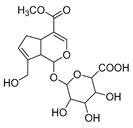	N	N		
**M12**	Sulfation	6.10	C_11_H_13_O_8_S	305.03365	305.03357	3.296	**101(100)**, **123(63)**, **69(39)**, **305(27)**, 96(15), **147(14)**, **225(13)**,**207(13)**	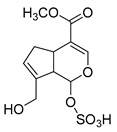	N	N	N	
**M15**	Demethylation	6.29	C_10_H_9_O_4_	193.05069	193.04991	1.941	**133(100)**, **178(53)**, **193(22)**,** 149(19)**, 137(17), 74(7.7), **109(1.9)**	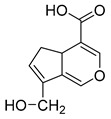			N	12 h(N), 24 h(N), 36 h(N), 48 h(N)
**M16**	Amination	6.43	C_11_H_16_O_4_N	226.10738	226.10699	−1.745	**194(100)**, **151(96)**, **226(48)**, 106(34), 80(25), 82(23), **120(22)**, 114(22), **148(20)**, 55(17),101(10),122(13),** 180(14)**, 79(10)	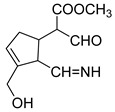	P		P	12 h(P), 24 h(P), 36 h(P), 48 h(P)
**M17**	Dehydrogenation and demethylation	6.45	C_10_H_9_O_5_	209.04549	209.04498	2.536	**121(100)**, 62(15), **209(11)**, 120(4.5), **181(3.1)**, 91(1.7), 79(1.4), **69(1.0), 136(1.0)**	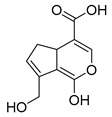		N	N	12 h(N), 24 h(N), 36 h(N), 48 h(N), 60 h(N)
**M21**	Decarbonylation	6.82	C_10_H_13_O_4_	197.08195	197.08128	2.256	**153(100)**,** 197(48**), **135(38)**, 59(24), **111(16)**, 83(7.6),** 69(7.2)**, **179(7.0)**, **123(5.9)**	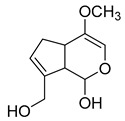	N			12 h(N), 24 h(N), 36 h(N), 48 h(N)
**M23**	Dehydroxylation	6.89	C_11_H_15_O_3_	195.10265	195.10201	2.521	**151(100)**, **95(71)**, 135(53), **195(39)**, **149(5.8)**, **80(3.2)**, **177(2.9)**, 130(2.5),**165(1.6)**	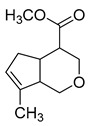			N	12 h(N), 24 h(N), 36 h(N), 48 h(N), 60 h(N)
**M25**	Glucuronidation	7.35	C_17_H_25_O_10_	389.14480	389.14548	3.229	**80(100)**,** 389(50)**, **81(34)**, **113(34)**, 85(25), **59(15)**, 71(15), 180(14), **235(13)**,73(10), **213(7.9)**	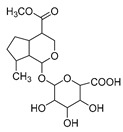	N			12 h(N), 24 h(N), 36 h(N), 48 h(N), 60 h(N)
**M28**	Decarbonylation	7.39	C_11_H_13_O_4_	197.08195	197.08119	1.799	**153(100)**, 59(46), 74(45), **135(12)**, 75(12), 137(7), **197(5.1)**, **179(0.99)**	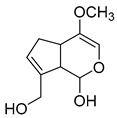	N			12 h(N), 24 h(N), 36 h(N), 48 h(N), 60 h(N)
**M29**	Glucuronidation	7.43	C_17_H_25_O_10_	389.14480	389.14532	2.818	**80(100)**, **389(51)**, 81(32),** 113(16), 235(14)**, 85(13), 180(12), 59(9), **213(5.6)**	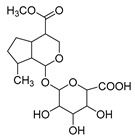	N			12 h(N), 24 h(N), 36 h(N), 48 h(N), 60 h(N)
**M30**	Aldehyde	7.45	C_11_H_13_O_5_	225.07685	225.07642	2.977	**225(100)**, **59 (44)**, **207(24)**,** 137(23)**, **163(19)**, **69(15)**, 109(12), **181(10)**	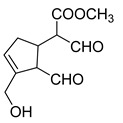	N		N	12 h(N), 24 h(N), 36 h(N), 48 h(N), 60 h(N)
**M32**	Oxygen-containing heterocycle	7.76	C_10_H_15_O_4_	199.09765	199.09765	0.515	**155(100)**, **199(60)**,** 137(37)**, 83(24), **181(14)**, **59(11)**, **130(4.9)**	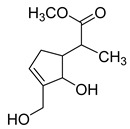	N		N	12 h(N), 24 h(N), 36 h(N), 48 h(N), 60 h(N)
**M34**	Dehydrogenation	8.00	C_11_H_11_O_5_	223.06115	223.06070	2.690	**223(100)**, **179(99)**, **155(37)**, 59(30), **161(12)**, **143(11)**, **195(8)**, 144(6.5)	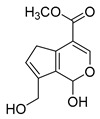	N			12 h(N), 24 h(N), 36 h(N), 48 h(N), 60 h(N)
**M35**	Dehydroxylation and hydrogenation	8.04	C_11_H_15_O_4_	211.09819	211.09695	2.201	**167(100)**,** 211(58)**, 81(49), 80(49), **132(29)**, **149(20)**, **59(15)**, 106(15), **99(1.0)**	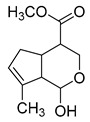	N			12 h(N), 24 h(N), 36 h(N), 48 h(N), 60 h(N)
**M37**	Dehydroxylation and hydrogenation	8.18	C_11_H_15_O_4_	211.09819	211.09697	0.485	**167(100)**, **59(14)**, 80(14), **149(13)**, 81(13), **211(10)**, **132(4.6)**, **99(1.2)**	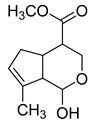	N			12 h(N), 24 h(N), 36 h(N), 48 h(N), 60 h(N)
**M38**	Deoxygenation and demethylation	8.21	C_11_H_15_O_2_	179.10775	179.10689	0.234	**179(100)**, 59(50), **135(21)**, 91(14), **161(11)**, 71(10)	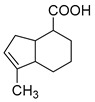	N		N	24 h(N), 36 h(N), 48 h(N), 60 h(N)
**M39**	Dehydroxylation and hydrogenation	8.27	C_11_H_15_O_4_	211.09819	211.09694	0.455	**99(100)**, **167(75)**, 80(18),** 211(13)**, **59(10)**, **149(9)**, **132(8.7)**	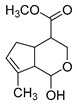	N			12 h(N), 24 h(N), 36 h(N), 48 h(N), 60 h(N)
**M40**	3-Methyl-genipin	8.30	C_12_H_15_O_5_	239.09255	239.09215	3.317	**239(100)**, **59(24)**,** 221(22)**, 83(18), **177(17)**, **151(12)**, **195(10)**,** 123(6.7)**	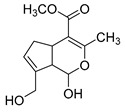	N			12 h(N), 24 h(N), 36 h(N), 48 h(N)
**M41**	Hydrogenation	8.32	C_11_H_17_O_4_	213.11270	213.11269	0.554	**169(100)**, **213(53)**, **151(23)**, **195(4)**, **73(2.8)**, 83(1.7), **113(1.5)**	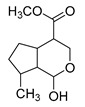			N	12 h(N), 24 h(N), 36 h(N), 48 h(N), 60 h(N)
**M43**	Hydrogenation	8.43	C_11_H_17_O_4_	213.11270	213.11270	0.564	**169(100)**, **213(27)**, **151(19)**, **73(2.0)**,**195(1.7)**, 170(1.4)	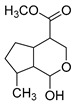			N	12 h(N), 24 h(N), 36 h(N), 48 h(N), 60 h(N)
**M44**	Hydrogenation	8.53	C_11_H_17_O_4_	213.11270	213.11269	0.554	**213(100)**, **169(92)**,** 151(56)**, **195(20)**, 97(10), 83(8.8),** 59(7.0)**, **113(3.3)**, **73(1.4)**	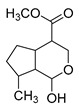			N	12 h(N), 24 h(N), 36 h(N), 48 h(N), 60 h(N)
**M45**	Hydrogenation	8.69	C_11_H_17_O_4_	213.11270	213.11272	0.584	**213(100)**, **169(92)**, **151(56)**, **195(20)**, 97(10), 83(8.8), **59(7.0),** 123(6.5), **73(1.7)**	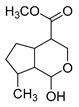			N	12 h(N), 24 h(N), 36 h(N), 48 h(N), 60 h(N)
**M46**	Dehydroxylation	8.82	C_11_H_13_O_4_	209.08087	209.08057	−1.269	**121(100)**, 93(63), **149(56)**, 91(47), 103(37), 79(26), **95(20)**, 85(19), **81(18)**, 105(16), **177(15)**, **131(13)**, 53(11), **209(7.7)**	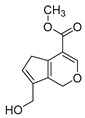		P	P	12 h(P), 24 h(P), 36 h(P), 48 h(P), 60 h(P)
**M48**	Glucuronic acid	9.45	C_15_H_19_O_10_	359.09839	359.09708	−0.538	**297(100)**, **82(86)**, 215(70), **171(70)**, **359(48)**, 81(27), **143(24)**,**125(24)**, 163(15)	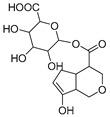			N	48 h(N), 60 h(N)
**M49**	3-Methyl-genipin	9.59	C_12_H_15_O_5_	239.09255	239.09219	3.304	**195(100)**, **151(69)**, **195(41)**, **239(33)**, **177(13)**, **59(7.1)**, **123(4.5)**	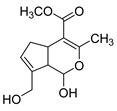	N			12 h(N), 24 h(N), 36 h(N), 48 h(N), 60 h(N)

P: positive ion mode. N: negative ion mode.

**Table 2:** Summary of genipinine as the metabolic center detected in vivo metabolites and in vitro fecal fermentation at different times.

Peak	Product	t_R_ /min	Formula	Theoretical Mass (*m/z*)	Experimental Mass (*m/z*)	Error (ppm)	MS/MS Fragment Ions	Identification	Vivo			Vitro
									U	B	F	Different Time (h)
**M1**	3-Hydroxy-3-(methylamino)propanoic acid	0.81	C_4_H_10_O_3_N	120.06567	120.06549	−0.247	**120(100)**, **103(53)**, 93(11), **91(5.9)**, 95(2), 53(1.6), **106(1.2)**	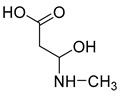		P		0 h(P), 12 h(P), 24 h(P), 36 h(P), 48 h(P), 60 h(P)
**M2**	5-(Hydroxymethyl) nicotinic acid	0.92	C_7_H_8_O_3_N	154.05007	154.04953	−2.205	**154(100)**, 92(21), **136(13)**, 112(8.3), **108(1.1)**, 95(3.0), **110(1.5)**	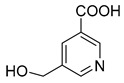			P	12 h(P), 24 h(P), 36 h(P), 48 h(P), 60 h(P)
**M5**	Hydroxylation	1.06	C_11_H_12_O_4_N	222.07602	222.07555	−0.534	**222(100)**, **134(34)**, **190(29)**, 176(28), **162(26)**, **144(13)**, 177(12), 106(7.0), **204(5.4)**	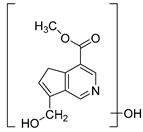	P			12 h(P), 24 h(P), 36 h(P), 48 h(P)
**M7**	Demethylation and dehydrogenation	4.86	C_10_H_12_O_3_N	194.08112	194.08081	−0.360	**91(100)**, **76(23)**, **57(1.9)**, 146(1.8), **134(1.8)**, 130(1.8), **108(1.7)**, **67(1.6)**, **194(1.0)**	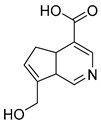	P		P	12 h(P), 24 h(P), 36 h(P), 48 h(P), 60 h(P)
**M8**	Dehydrogenation	5.33	C_11_H_12_O_3_N	206.08107	206.08069	−2.328	**146(100)**, **174(64)**, **206(58)**, **118(44)**, **91(25)**, 154(19), **178(10)**, **156(7.7)**	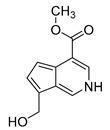			p	12 h(P), 24 h(P), 36 h(P), 48 h(P), 60 h(P)
**M10**	Demethylation and dehydrogenation	5.48	C_10_H_12_O_3_N	194.08112	194.08086	−0.310	**91(100)**, **76(23)**, **134(7.4)**, **194(3.5)**, **150(3.4)**, 106(3.1), 57(2.2), 92(2.1), 130(2.0)	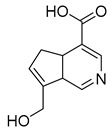	P		P	12 h(P), 24 h(P), 36 h(P), 48 h(P), 60 h(P)
**M13**	Dehydrogenation	6.12	C_11_H_14_NO_3_	208.09682	208.09636	−2.210	**120(100)**, 85(21), **166(6.4)**, 103(6.0), **162(2.4)**, **148(1.7)**, **208(1.5)**	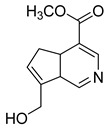	P		P	12 h(P), 24 h(P), 36 h(P), 48 h(P), 60 h(P)
**M14**	Hydrogenation	6.20	C_9_H_12_O_4_N	198.07617	198.07578	−1.537	**138(100)**, **123(32)**, 96(19), **126(15)**, **110(12)**, 120(12), 130(10), 55(9.4), **108(2.6)**	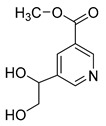	P			12 h(P), 24 h(P), 36 h(P), 48 h(P)
**M18**	Dehydrogenation	6.49	C_11_H_12_O_3_N	206.08107	206.08080	−1.794	**118(100)**, **130(68)**, **146(37)**, **170(34)**, **188(27)**, **160(23)**,** 132(22)**, **206(18)**, **142(11)**	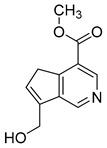	P			12 h(P), 24 h(P), 36 h(P), 48 h(P), 60 h(P)
**M19**	Glycine conjugation	6.56	C_13_H_19_O_4_N_2_	267.13397	267.13339	−2.035	**134(100)**, 162(17),** 161(15)**, 84(13), 197(6.6), **239(5.5)**, **135(5.4)**, 80(3.1),**130(2.4)**, **207(1.9)**	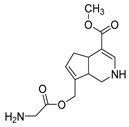	P			12 h(P), 24 h(P), 36 h(P), 48 h(P), 60 h(P)
**M20**	Carbonylation	6.66	C_11_H_14_O_4_N	224.09167	224.09128	−2.208	**146(100)**, **192(54)**, **118(53)**, **82(48)**, **178(42)**, 174(36), 114(32), **224(31)**, **206(26),** 91(19), 105(12), 60(11)	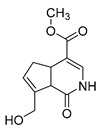	P			12 h(P), 24 h(P), 36 h(P), 48 h(P), 60 h(P)
**M22**	Demethylation and hydroxylation	6.88	C_10_H_14_O_4_N	212.09167	212.09132	−1.954	**109(100)**, 76(15), **137(1.7), 81(1.7)**, 95(1.5), 79(1.3), **166(1.0)**,134(1.0)	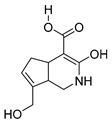		P		12 h(P), 24 h(P), 36 h(P), 48 h(P), 60 h(P)
**M24**	Dehydrogenation	7.00	C_11_H_12_O_3_N	206.08107	206.08084	−1.600	**118(100)**, **130(68)**, **146(37)**, **170(34)**, **95(1.4)**, **146(1.2)**, **116(1.1)**, **206(1.1)**	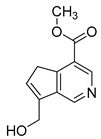	P			12 h(P), 24 h(P), 36 h(P), 48 h(P), 60 h(P)
**M26**	Dehydrogenation	7.35	C_11_H_10_O_3_N	204.06542	204.06512	−0.400	**204(100)**, **144(71)**, **116(50)**, **92(29)**, 134(15), **65(14)**, 120(12), 176(11), **175(4.7)**	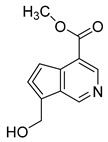	P		P	12 h(P), 24 h(P), 36 h(P), 48 h(P), 60 h(P)
**M27**	Sulfation	7.36	C_11_H_16_O_7_NS	306.06417	306.06418	−0.062	**147(100)**, **81(60)**, 289(43), 119(42), 105(40), **175(38)**, 157(30), **67(28)**, 165(27), **123(23)**, **210(15)**	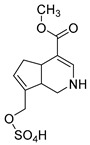	P			48 h(P)
**M31**	Genipinine	7.72	C_11_H_16_O_3_N	210.11247	210.11214	−1.457	**178(100)**, **210(36)**, 109(32), 81(21), 79(17), 84(12), **132(12)**, **160(9)**, 133(8.8)	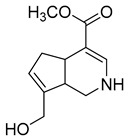			P	12 h(P), 24 h(P), 36 h(P), 48 h(P)
**M33**	Hydroxylation	7.83	C_11_H_12_O_4_N	222.07602	222.07562	−0.464	**119(100)**, **204(70)**, **222(37)**, **162(34)**, **144(31)**, **116(27)**, 118(26), 172(23), **105(19)**	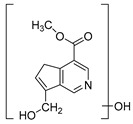	P			12 h(P), 24 h(P), 36 h(P), 48 h(P)
**M36**	Hydrogenation	8.06	C_11_H_20_O_3_N	214.14322	214.14326	−2.382	**70(100)**, **116(68)**, 81(38), **71(10)**, **214(4.1)**, 99(4.04), 79(3.5), **168(2.4)**	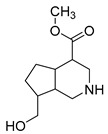	P		P	12 h(P), 24 h(P), 36 h(P), 48 h(P), 60 h(P)
**M42**	Dehydroxylation	8.32	C_11_H_12_O_2_N	190.08632	190.08589	−0.365	**130(100)**, **55(21)**,** 172(8.1)**, **190(6.0)**, 149(3.4), 144(1.5), 72(1.4),** 162(1.2)**	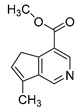			P	12 h(P), 24 h(P), 36 h(P), 48 h(P), 60 h(P)
**M47**	Dehydroxylation and glucuronidation	9.40	C_16_H_22_O_8_N	356.13402	356.13348	−0.508	**135(100)**,** 93(40)**, **107(38)**, **356(35),** 67(15), 109(15), 60(12), **180(5.6)**	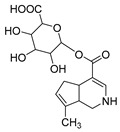	P			48 h(P)

P: positive ion mode. N: negative ion mode.

## Data Availability

Most of the data used during the preparation of the manuscript are included in the Results and Discussion sections. However, for any additional details of the procedures and the original raw files, please contact the corresponding authors.

## Supplementary Materials

The following supporting information can be downloaded at: https://www.mdpi.com/article/10.3390/molecules28176307/s1. Figure S1: Identification diagram of genipin metabolites; Table S1: Classification of metabolites in fecal fermentation.

## Sample Availability

Samples of the related compounds are available from the authors.
