# Supplementary material for: Comprehensive Metabolite Identification of Genipin in Rats Using Ultra-High-Performance Liquid Chromatography Coupled with High Resolution Mass Spectrometry
Source: Molecules. 2023 Aug 29;28(17):6307. doi: 10.3390/molecules28176307 (PMC10489007; doi:10.3390/molecules28176307)
Supplement: Supplementary file 1 [file molecules-28-06307-s001.zip › molecules-2561541-supplementary.pdf]

Article

Comprehensive Metabolite Identification of Genipin in Rats Using Ultra-High-Performance Liquid Chromatography
Coupled with High Resolution Mass Spectrometry

Zhifeng Cui [1,2,†], Zhe Li [3,†], Weichao Dong [3,†], Lili Qiu [4], Jiayu Zhang [1,*] and Shaoping Wang [1,*]

Supplementary Figure S1. Identification diagram of genipin metabolites.

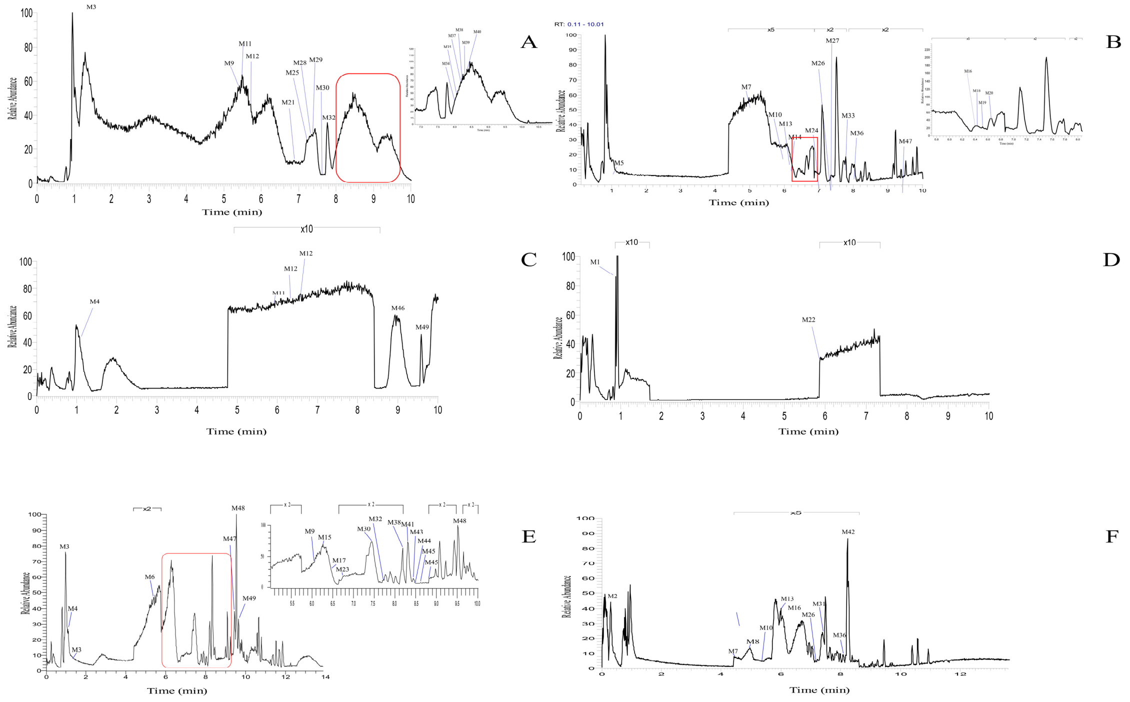

Supplementary Table S1. Classification of metabolites in fecal fermentation.

Metabolites	0h	12h	24h	36h	48h	60h
**M0**	√	√	√	√	√	√
**M1**	√	√	√	√	√	√
**M2**	×	√	√	√	√	√
**M3**	×	×	×	×	×	×
**M4**	√	√	√	√	√	√

**M5**	×	√	√	√	√	×
**M6**	×	√	√	√	√	×
**M7**	×	√	√	√	√	√
**M8**	×	√	√	√	√	√
**M9**	×	×	×	×	×	×
**M10**	×	√	√	√	√	√
**M11**	×	×	×	×	×	×
**M12**	×	×	×	×	×	×
**M13**	×	√	√	√	√	√
**M14**	×	√	√	√	√	×
**M15**	×	√	√	√	√	×
**M16**	×	√	√	√	√	×
**M17**	×	√	√	√	√	√
**M18**	×	√	√	√	√	√
**M19**	×	√	√	√	√	√
**M20**	×	√	√	√	√	√
**M21**	×	√	√	√	√	×
**M22**	×	√	√	√	√	√
**M23**	×	√	√	√	√	√
**M24**	×	√	√	√	√	√
**M25**	×	√	√	√	√	√
**M26**	×	√	√	√	√	√
**M27**	×	×	×	×	√	×
**M28**	×	√	√	√	√	√
**M29**	×	√	√	√	√	√
**M30**	×	√	√	√	√	√
**M31**	×	√	√	√	√	×
**M32**	×	√	√	√	√	√
**M33**	×	√	√	√	√	×

**M34**	×	√	√	√	√	√
**M35**	×	√	√	√	√	√
**M36**	×	√	√	√	√	√
**M37**	×	√	√	√	√	√
**M38**	×	×	√	√	√	√
**M39**	×	√	√	√	√	√
**M40**	×	√	√	√	√	×
**M41**	×	√	√	√	√	√
**M42**	×	√	√	√	√	√
**M43**	×	√	√	√	√	√
**M44**	×	√	√	√	√	√
**M45**	×	√	√	√	√	√
**M46**	×	√	√	√	√	√
**M47**	×	×	×	×	√	×
**M48**	×	×	×	×	√	√
**M49**	×	√	√	√	√	√
